# Therapie der sepsisinduzierten Koagulopathie: Ergebnisse einer deutschlandweiten Umfrage auf Intensivstationen

**DOI:** 10.1007/s00101-021-00916-9

**Published:** 2021-02-08

**Authors:** Thomas Schmoch, Thorsten Brenner, Andrea Becker-Pennrich, Ludwig Christian Hinske, Markus A. Weigand, Josef Briegel, Patrick Möhnle, SepNet Study Group

**Affiliations:** 1 Klinik für Anästhesiologie, Universitätsklinikum Heidelberg, Heidelberg, Deutschland; 2 Klinik für Anästhesiologie und Intensivmedizin, Universitätsklinikum Essen, Hufelandstraße 55, 45147 Essen, Deutschland; 3 Klinik für Anästhesiologie und Abteilung für Transfusionsmedizin, Zelltherapeutika und Hämostaseologie, LMU Klinikum München, München, Deutschland; 4 Institut für medizinische Informationsverarbeitung, Biometrie und Epidemiologie, LMU München, München, Deutschland

**Keywords:** Thromboseprophylaxe, Antikoagulation, Sepsis, Covid-assoziierte Koagulopathie, COVID-19, Thromboembolism, Anticoagulation, Sepsis, Covid-associated Coagulopathy, COVID-19

## Abstract

**Hintergrund:**

Im Rahmen von Sepsis und septischem Schock kommt es aufgrund der engen Verflechtung von Gerinnung und Entzündung häufig zu einer Koagulopathie. Die sepsisinduzierte Koagulopathie (SIC) stellt hierbei die schwerste, potenziell fatale Form dar. Aufgrund fehlender Evidenz beschränken sich die aktuellen Sepsis-Leitlinien auf Empfehlungen zur medikamentösen Prophylaxe einer venösen Thromboembolie (VTE), während die Behandlung einer SIC nicht thematisiert wird.

**Methoden:**

Um den Status quo der VTE-Prophylaxe sowie der SIC-Behandlung auf deutschen Intensivstationen zu erheben, wurde von Oktober 2019 bis Mai 2020 eine deutschlandweite Onlineumfrage unter ärztlichen Leitern von Intensivstationen durchgeführt. Diese wurde ab April 2020 durch einen zusätzlichen Fragenblock ergänzt, der sich mit der VTE-Prophylaxe sowie der SIC-Behandlung bei Coronaviruskrankheit(COVID)-19-Patienten befasste.

**Ergebnisse:**

Die Umfrageergebnisse zeigen eine ausgeprägte Heterogenität in der klinischen Praxis bezüglich Prophylaxe von VTE und Therapie der SIC. Ein systematisches Screening auf SIC findet in den meisten Intensivstationen nicht statt. Bei COVID-19-Patienten fällt v. a. auf, dass bei drei Viertel der teilnehmenden Intensivstationen die gelebte Praxis der medikamentösen VTE-Prophylaxe nicht von Non-COVID-19-Patienten abweicht.

**Schlussfolgerung:**

Die Heterogenität der in der Umfrage gesammelten Antworten legt nahe, dass es einer systematischen Aufarbeitung dieses Themenfeldes bedarf, um zukünftig über klinische Interventionsstudien die individualisierte Patientenversorgung mit der gebotenen Evidenz zu unterlegen.

**Zusatzmaterial online:**

Die Online-Version dieses Beitrags (10.1007/s00101-021-00916-9) enthält eine Zusammenstellung weiterer Aspekte zum Beitrag und den zugrunde liegenden Fragebogen. Beitrag und Zusatzmaterial stehen Ihnen auf www.springermedizin.de zur Verfügung. Bitte geben Sie dort den Beitragstitel in die Suche ein, das Zusatzmaterial finden Sie beim Beitrag unter „Ergänzende Inhalte“.

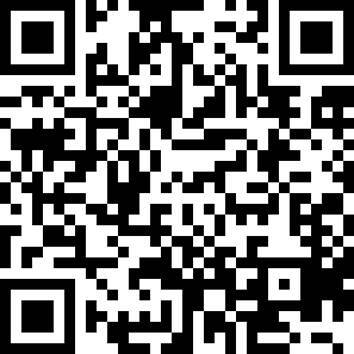

## Abstract (en)

**Background:**

In the context of sepsis and septic shock, coagulopathy often occurs due to the close relationship between coagulation and inflammation. Sepsis-induced coagulopathy (SIC) is the most severe and potentially fatal form. Anticoagulants used in prophylactic or therapeutic doses are discussed to potentially exert beneficial effects in patients with sepsis and/or SIC; however, due to the lack of evidence recent guidelines are limited to recommendations for drug prophylaxis of venous thromboembolism (VTE), while treatment of SIC has not been addressed.

**Methods:**

In order to determine the status quo of VTE prophylaxis as well as treatment of SIC in German intensive care units (ICU), we conducted a Germany-wide online survey among heads of ICUs from October 2019 to May 2020. In April 2020, the survey was supplemented by an additional block of questions on VTE prophylaxis and SIC treatment in coronavirus disease 2019 (COVID-19) patients.

**Results:**

A total of 67 senior doctors took part in the survey. The majority (*n* = 50; 74.6%) of the responses were from ICU under the direction of an anesthesiologist and/or a department of anesthesiology. Most of the participants worked either at a university hospital (*n* = 31; 47.8%) or an academic teaching hospital (*n* = 27; 40.3%). The survey results show a pronounced heterogeneity in clinical practice with respect to the prophylaxis of VTE as well as SIC treatment. In an exemplary case of pneumogenic sepsis, low molecular weight heparins (LMWH) were by far the most frequently mentioned group of medications (*n* = 51; 76.1% of the responding ITS). In the majority of cases (*n* = 43; 64.2%), anti-FXa activity is not monitored with the use of LMWH in prophylaxis doses. Unfractionated heparin (UFH) was listed as a strategy for VTE prophylaxis in 37.3% of the responses (*n* = 25). In an exemplary case of abdominal sepsis 54.5% of the participants (*n* = 36; multiple answers possible) stated the use of UFH or LMWH and UFH with dosage controlled by PTT is used on two participating ICUs. The anti-FXa activity under prophylactic anticoagulation with LMWH is monitored in 7 participating clinics (10.6%) in abdominal sepsis. Systematic screening for sepsis-associated coagulation disorders does not take place in most hospitals and patterns in the use of anticoagulants show significant variability between ICUs. In the case of COVID-19 patients, it is particularly noticeable that in three quarters of the participating ICUs the practice of drug-based VTE prophylaxis and SIC treatment does not differ from that of non-COVID-19 patients.

**Conclusion:**

The heterogeneity of answers collected in the survey suggests that a systematic approach to this topic via clinical trials is urgently needed to underline individualized patient care with the necessary evidence.

## Hintergrund und Fragestellung

Sepsis und septischer Schock sind die schwerstmöglichen Verlaufsformen einer Infektion [22]. Aufgrund der physiologischen Interaktion von Inflammation und Koagulation [6] sind Störungen des Gerinnungssystems als fester Bestandteil der Pathophysiologie der Sepsis zu verstehen. In den letzten Jahren hat sich der Begriff sepsisinduzierte Koagulopathie (SIC) etabliert [10, 11, 13]. Die SIC definiert die Maximalform einer disseminierten intravasalen Koagulopathie (DIC) auf dem Boden einer Sepsis; abgegrenzt wird diese von einer DIC anderer Genese (z. B. Pankreatitis oder Verbrennung; Zusatzmaterial online: Zusammenstellung – Tab. 1) [10]. Bis heute sind nur wenige Studien durchgeführt worden, die erstens den Nutzen einer medikamentösen Prophylaxe einer venösen Thromboembolie (VTE) bei Intensivpatienten und zweitens den Nutzen einer therapeutischen Antikoagulation bei Patienten mit Sepsis untersuchen. Zwar wird eine medikamentöse VTE-Prophylaxe mit niedermolekularem Heparin (NMH) oder unfraktioniertem Heparin (UFH) allgemein für Patienten in intensivmedizinischer Behandlung [4] und damit auch für Patienten mit Sepsis und septischem Schock [1, 20] empfohlen. Ob jedoch Patienten, die eine SIC entwickeln, von einer, über die VTE-Prophylaxe hinausgehenden, therapeutischen Antikoagulation profitieren könnten, ist wissenschaftlich kaum untersucht und in nationalen und internationalen Leitlinien nicht abgebildet [1, 20]. Gleiches gilt für das Vorgehen bei Patienten, die an einer „coronavirus disease 2019“ (COVID-19) erkrankt sind, da insbesondere schwere Verläufe dieser Erkrankung mit einer ausgeprägten Koagulopathie und einem deutlich erhöhten Risiko für thrombembolische Ereignisse im Sinne einer „COVID-19-associated coagulopathy“ (CAC) assoziiert sind [9, 16]. Während internationale Leitlinien weitgehend an einer „normalen“ VTE-Prophylaxe festhalten, geht die Ende November 2020 veröffentlichte S2K-Leitlinie zur stationären Therapie von COVID-19-Patienten einen Schritt weiter und empfiehlt bei intensivpflichtigen Patienten mit COVID-19 eine „intensivierte“ VTE-Prophylaxe [15, 25].

Die Folge dieser unklaren Studienlage ist, dass intensivmedizinische Bereiche jeweils eigene Konzepte entwickeln, um die VTE-Prophylaxe sowie die therapeutische Antikoagulation für den klinischen Alltag in ihrem Bereich zu standardisieren und dabei gleichzeitig der Heterogenität der Patienten und deren individuellen Bedürfnissen gerecht zu werden. Die vorliegende Umfrage soll Einblicke in die auf deutschen Intensivstationen gelebte Praxis der prophylaktischen und therapeutischen Antikoagulation bei intensivmedizinischen Patienten allgemein, bei Patienten mit Sepsis und septischem Schock (mit/ohne SIC) sowie bei Patienten mit COVID-19 geben.

## Studiendesign und Untersuchungsmethoden

Grundlage unserer Erhebung war eine deutschlandweite Onlineumfrage zwischen dem 01.10.2019 und dem 30.05.2020. Der Fragebogen (Zusatzmaterial online: Fragebogen) richtete sich gezielt an die *ärztlichen Leiter einer Intensivstation (ITS)* oder eines Intensivbereiches mit dem explizit formulierten Ziel, nur einen ausgefüllten Fragebogen pro Intensivbereich zu erhalten, ohne dass eine doppelte Teilnahme technisch blockiert war. Der Link zu dem Fragebogen wurde zunächst über die Kliniken der SepNet Study Group verteilt. In einem zweiten Schritt wurden im April 2020 alle in der Deutschen Interdisziplinären Vereinigung für Intensiv- und Notfallmedizin (DIVI) organisierten intensivmedizinischen Abteilungen sowie die Mitglieder der „Interdisziplinären Arbeitsgemeinschaft für Klinische Hämotherapie“ (IAKH) per E‑Mail zur Teilnahme an der Umfrage eingeladen. Die Datenbank wurde am 31.05.2020 geschlossen. Die Teilnahme erfolgte vollständig anonym.

Der Onlinefragebogen wurde nach ausführlicher Literaturrecherche und in enger Rücksprache mit den ausgewiesenen Experten der SepNet Study Group erstellt. Einfach- und Mehrfachauswahlfragen sowie Freitextfelder wurden zur Erhebung genutzt.

Der Fragebogen umfasste 5 Fragenkomplexe zu „Infrastruktur“, „Status quo Antikoagulation“, „Status quo Sepsis“, „Status quo sepsisassoziierte DIC“ und „COVID-19“. Kern des Fragebogens waren 2 Fallvignetten von Patienten mit pneumogener Sepsis (Zusatzmaterial online: Zusammenstellung – Fallbeispiel 1) bzw. abdomineller Sepsis bei sekundärer Peritonitis und erfolgter chirurgischer Fokussanierung (Zusatzmaterial online: Zusammenstellung – Fallbeispiel 2). Nachfolgend wurde in Variationen der Fälle abgefragt, wie die Teilnehmer entsprechende Patienten in Bezug auf die VTE-Prophylaxe behandeln bzw. therapeutisch antikoagulieren würden.

Der letzte Teil des Fragebogens, der sich mit den Besonderheiten bei COVID-19 befasst, wurde im April 2020 online gestellt. Somit konnte nur ein Teil der Teilnehmer die Fragen zu COVID-19 beantworten.

Eine ausführlichere Darstellung der Methodik ist in den Internet-Supplements (Zusatzmaterial online: Zusammenstellung – Methodenteil) zu finden.

### Statistik

Zur statistischen Auswertung wurden deskriptive Methoden mittels Microsoft® Office Excel (Excel für Mac Version 16.3; Fa. Microsoft Corporation, Redmond, WA, USA) und Prism® 8 for macOS, (GraphPad Prism® für Mac Version 8.3.0; Fa. GraphPad Software LLC, San Diego, CA, USA) verwendet. Es wurden absolute und relative Häufigkeiten dargestellt. Das arithmetische Mittel, Median und Quartile wurden berechnet, wenn immer es sinnvoll erschien.

## Ergebnisse

### Charakteristika der teilnehmenden Kliniken

Insgesamt nahmen 67 leitende Ärztinnen und Ärzte an der Umfrage teil. Der größte Teil (*n* = 50; 74,6 %) der Antworten kam von anästhesiologisch geleiteten ITS, und die meisten Teilnehmer arbeiteten entweder an einem Universitätsklinikum (*n* = 31; 47,8 %) oder einem akademischen Lehrkrankenhaus (*n* = 27; 40,3 %) (Tab. 1). Die teilnehmenden ITS hatten im Median 16 Beatmungsbetten (12,0–27,0; 25.–75. Perzentil) mit einem medianen Beatmungsanteil von 55 % (33–70 %, 25.–75. Perzentil); die mediane Verweildauer lag bei 5 Tagen (4 bis 7 Tage; 25.–75. Perzentil).

**Table 1:** 

	Alle Fragebogen		Fragebogen mit Beantwortung des COVID-19 Fragenanteils	
	***n***	**%**	***n***	**%**
*Insgesamt beantwortete Fragebogen*	67	–	31	–
*Fachdisziplin der ITS-Leitung*				
Anästhesiologie	50	74,6	20	64,5
Allgemein- und Viszeralchirurgie	3	4,5	2	6,5
Pneumologie	2	3,0	0	0
Allg. innere Medizin	8	11,9	6	19,4
Pädiatrie	1	1,5	1	3,2
Interdisziplinär	3	4,5	2	6,5
*Fachdisziplin der Patienten, die auf der ITS behandelt werden*				
Allgemein- und Viszeralchirurgie	59	88,1	28	90,3
Neurochirurgie	31	46,3	10	32,3
Traumatologie und Orthopädie	55	82,1	26	83,9
Herzchirurgie	21	31,3	6	19,4
Gefäßchirurgie	44	65,7	18	58,1
Thoraxchirurgie	37	55,2	13	41,9
Gynäkologie	41	61,2	20	64,5
Urologie	52	77,6	24	77,4
Pulmonologie	38	56,7	21	67,7
Kardiologie	38	56,7	22	71,0
Gastroenterologie und Hepatologie	45	67,2	26	83,9
Neurologie	38	56,7	18	58,1
Sonstige	8	11,9	3	9,7
*Multidisziplinär*	65	97,0	31	100
*Nur eine Fachrichtung*	2	3,0	0	0
*Bettenzahl des Krankenhauses*				
<100	1	1,5	0	0
100–250	5	7,5	4	12,9
251–500	16	23,9	11	35,5
501–1000	16	23,9	7	22,6
>1000	28	41,8	9	29,0
K. A.	1	1,5	0	0
*Art des Krankenhauses, zu der die ITS gehört*				
Universitätsklinikum	32	47,8	10	32,3
Lehrkrankenhaus	27	40,3	16	51,6
Krankenhaus	6	9,0	4	12,9
K. A.	1	1,5	0	0
	**Mittelwert [d]**	**Median (25.–75. Perz.)**	**Mittelwert [d]**	**Median [d] (25.–75. Perz.)**
*Mittlere Verweildauer*	6,3	5,0 (4,0–7,0)	5,4	4,0 (4,0–7,0)
*Durchschnittlicher Anteil an invasiv beatmeten Patienten, (%)*	51,9	55,0 (33,0–70,0)	50,8	55,0 (40,0–65,3)
*Anzahl der Intensivbetten (nur Beatmungsbetten, keine IMC)*	22,8	16,0 (12,0–27,0)	18,3	14,5 (12,0–18,0)

*IMC* intermediate Care, *ITS* Intensivstation

### Verwendete SIC-Testverfahren

Auf den meisten ITS (*n* = 53; 79,1 %) wird nicht regelmäßig auf das Vorliegen einer DIC getestet. Lediglich 7,5 % (*n* = 5) berechnen den International Society on Thrombosis and Haemostasis (ISTH) Score [23], 6 % (*n* = 4) den Japanese Association for Acute Medicine Disseminated Intravascular Coagulation (JAAM DIC) Score [8], und 4,5 % (*n* = 3) gaben an, ein anderes Testverfahren zu nutzen. Drei Viertel der Teilnehmer gaben an, zwar keine strukturierten Suchtests anzuwenden, aber bei klinischen Hinweisen auf eine DIC eine gezielte Behandlung einzuleiten (Tab. 2).

**Table 2:** 

	Alle Fragebogen		Fragebogen mit Beantwortung des COVID-19 Fragenanteils	
	*n*	%	*n*	%
*Screening auf sepsisassoziierte Koagulopathie (SIC)*				
Japanese Association for Acute Medicine Disseminated Intravascular Coagulation (JAAM DIC) Score	4	6,0	2	6,5
International Society on Thrombosis and Haemostasis (ISTH) Score	5	7,5	2	6,5
Anderer Score	3	4,5	3	9,7
Es wird nicht regelmäßig gescreent	53	79,1	23	74,2
K. A.	2	3,0	1	3,2
*Wenn Sie Anhaltspunkte für eine beginnende DIC haben, behandeln Sie diese?*				
Ja	51	76,1	24	77,4
Nein	9	13,4	3	9,7
K. A.	7	10,4	4	12,9

### Verfügbare Gerinnungsdiagnostik

**Figure 1:**
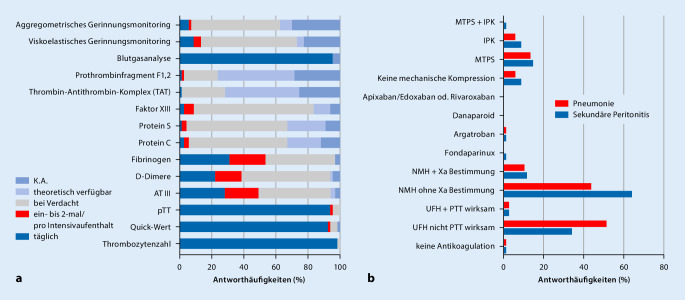

In nahezu allen teilnehmenden ITS werden neben der Blutgasanalyse, die Thrombozytenzahl, der Quick-Wert und die partielle Thromboplastinzeit (PTT) täglich bestimmt (Abb. 1a). Antithrombin (AT), D‑Dimere und Fibrinogen werden auf 20–30 % der ITS täglich bestimmt. Bei weiteren 20 % werden sie regelhaft im Laufe eines ITS-Aufenthaltes bestimmt, und fast alle befragten Einrichtungen nutzen sie zumindest in konkreten Verdachtsfällen. Im Gegensatz dazu werden die Aktivitäten von Protein C, Protein S und Faktor XIII in über 60 % der ITS nur in spezifischen Verdachtsfällen bestimmt, sind aber in fast allen Häusern zumindest theoretisch verfügbar. Auch viskoelastische (z. B. ROTEM®, Tem Innovations GmbH, München, Deutschland; TEG®, Haemonetics Cooperation, Boston, MA, USA) und aggregometrische (z. B. Multiplate®, F. Hoffmann-La Roche AG, Basel, Schweiz; ROTEM delta platelet®, Tem Innovations GmbH, München, Deutschland) Verfahren des Gerinnungsmonitorings sind auf über 60 % der ITS verfügbar und kommen regelhaft im Verdachtsfall zum Einsatz. Hierbei gibt es allerdings ein starkes Gefälle der Verfügbarkeit von universitären Häusern über Lehrkrankenhäusern zu kleineren Krankenhäusern (Zusatzmaterial online: Zusammenstellung – Abb. 1).

### Medikamentöse VTE-Prophylaxe bei Sepsis

Im Rahmen der Fallvignetten wurde nach der VTE-Prophylaxe bei Patienten mit pneumogener Sepsis und abdomineller Sepsis (auf dem Boden einer sekundären Peritonitis) gefragt. In beiden Fällen wurde die prophylaktische Antikoagulation auf den meisten ITS mit niedermolekularem Heparin (NMH) oder mit unfraktioniertem Heparin (UFH) durchgeführt (Abb. 1b; Zusatzmaterial online: Zusammenstellung – Tab. 2). Im Falle der pneumogenen Sepsis war NMH die mit Abstand am häufigsten genannte Präparategruppe (*n* = 51; 76,1 % der antwortenden ITS). Dabei wird in der überwiegenden Mehrheit der Fälle (*n* = 43; 64,2 %) die Anti-FXa-Aktivität im Rahmen einer prophylaktischen Anwendung laborchemisch nicht kontrolliert. Unfraktioniertes Heparin wurde in 37,3 % der Antworten als Strategie zur VTE-Prophylaxe genannt (*n* = 25). Interessanterweise gaben 2 der teilnehmenden ITS an (3,0 %), in solchen Fällen eine PTT-wirksame VTE-Prophylaxe (ohne einheitliche Definition des Begriffes „PTT-wirksam“) durchzuführen. Im Falle der abdominellen Sepsis gaben jeweils 54,5 % der Teilnehmer (*n* = 36; Mehrfachantworten möglich) an, UFH oder NMH zu nutzen. Auf 2 ITS wird UFH dabei PTT-wirksam eingesetzt. Die Anti-FXa-Aktivität unter prophylaktischer Antikoagulation mit NMH wird bei abdomineller Sepsis in 7 Kliniken (10,6 %) regelhaft bestimmt.

Eine mechanische VTE-Prophylaxe mittels medizinischer Thromboseprophylaxestrümpfe (MTPS), intermittierender pneumatischer Kompression (IPK) sowie die Kombination der beiden genannten werden jeweils nur bei weniger als 10 % der teilnehmenden ITS regelhaft durchgeführt.

Mehrere Teilnehmer nutzten die Möglichkeit zur Eingabe freier Kommentare. Diese sind in den Internet-Supplements (Zusatzmaterial online: Zusammenstellung – Freitext 1) zum Nachlesen bereitgestellt.

### VTE-Prophylaxe und Antikoagulation bei SIC

**Figure 2:**
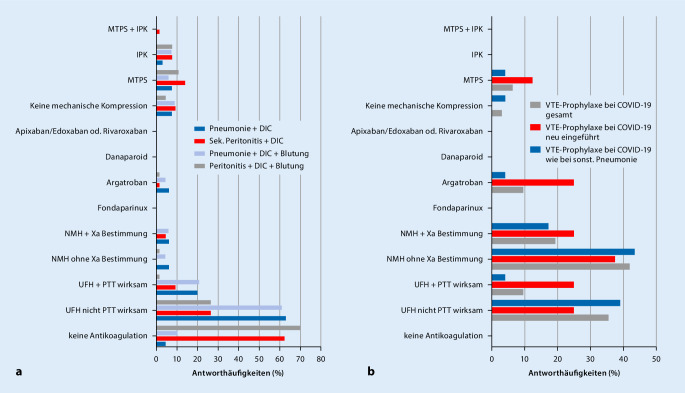

In Modifikationen der Fallvignetten von pneumogener Sepsis und abdomineller Sepsis wurden die Teilnehmenden gefragt, ob und, wenn ja, wie sie eine SIC behandeln würden, je nachdem, ob zusätzliche Blutungszeichen bestünden oder nicht. Hierbei unterschieden sich die Antworten deutlich: Bei pneumogener Sepsis mit SIC ohne Blutung würden nur 3 der teilnehmenden ITS (4,6 %) keine Antikoagulation durchführen oder die bestehende Antikoagulation absetzen. Die meisten Teilnehmenden (*n* = 41; 63,1 %) würden UFH in einer nicht-PTT-wirksamen Dosierung zur VTE-Prophylaxe verwenden (Abb. 2a; Zusatzmaterial online: Zusammenstellung – Tab. 2). 20,0 % der Teilnehmenden gaben an, UFH PTT-wirksam einzusetzen. NMH wird auf 4 ITS ohne und auf 4 ITS mit Anti-FXa-Monitoring eingesetzt (jeweils 6,2 %). Auch Argatroban gaben 4 Intensivstationen (6,2 %) als mögliche Option an. Fondaparinux, Danaparoid sowie Apixaban, Edoxaban und Rivaroxaban kommen nicht zur Anwendung. In den Freitextkommentaren zur pneumogenen Sepsis mit SIC ohne Blutung zeigte sich, dass die Definition einer niedrigdosierten Gabe von UFH deutlich zwischen den Kliniken variiert. So wurden 6‑mal Dosisangaben zwischen 100 und 500 I.E./h gemacht, 6 I.E./kgKG und h sowie 3‑mal PTT-Zielwerte < 40 s (ohne Dosisangaben) genannt. In einer Antwort wurden 500–800 I.E./h angegeben, um ein PTT-Ziel von 35 s zu erreichen, 2‑mal wurde ein PTT-Ziel von 40–50 s genannt, 2‑mal 50–60 s und einmal sogar 60–70 s. Argatroban (Ziel-PTT 40–60 s) kam einmal vor, ebenso Enoxaparin „40–60 mg/Tag i.v.“ und Enoxaparin „2-mal 1 mg/kgKG mit einer Ziel Anti-FXa-Aktivität von 0,4–0,8“ IU/ml.

Bei einer pneumogenen Sepsis mit SIC und diffuser Blutung wird auf 62,5 % der ITS die vorbestehende Antikoagulation abgesetzt, 26,6 % nutzen UFH nicht PTT-wirksam als VTE-Prophylaxe, und 9,4 % antikoagulieren PTT-wirksam mit UFH. NMH werden von 4,7 % der ITS eingesetzt, allerdings ausschließlich unter Anti-FXa-Aktivität-Monitoring. Argatroban nutzt in diesem Fall eine der teilnehmenden ITS. Bei der Behandlung einer DIC als Komplikation einer abdominellen Sepsis nach Fokussanierung würden 10,4 % keine Antikoagulation durchführen. Die Mehrzahl von 61,2 % spricht sich für eine VTE-Prophylaxe mittels UFH aus, und 20,9 % würden UFH PTT-wirksam einsetzen. Eine Aufschlüsselung der Daten bezüglich i.v.- oder s.c.-Gabe erfolgte nicht. Argatroban wird nur in 3 (4,5 %) Antworten als Option bei abdomineller Sepsis genannt. Danaparoid, Apixaban, Edoxaban und Rivaroxaban wurden von keinem der Antwortenden als Option benannt. Kommt es bei einer abdominellem Sepsis zu einer SIC mit diffuser Blutung, führen 45 (70,3 %) keine medikamentöse Antikoagulation mehr durch. 26,6 % gaben an, UFH nicht PTT-wirksam einzusetzen. NMH, Fondaparinux, Danaparoid, Argatroban und DOAK werden bei Blutungen nicht genutzt (Abb. 2a; Zusatzmaterial online: Zusammenstellung – Tab. 2).

Die Freitextantworten zu pneumogener Sepsis und abdomineller Sepsis mit SIC und mit und ohne Blutung sind in den Internet-Supplements nachzulesen (Zusatzmaterial online: Zusammenstellung – Freitexte 2 und 3).

### Umfrage COVID-19

Nachdem im Rahmen der SARS-CoV-2-Pandemie im Frühjahr 2020 deutlich wurde, dass Patienten, die an COVID-19 erkrankt sind, zu einer Hyperkoagulopathie im Sinne einer „COVID-19-associated coagulopathy“ (CAC) neigen [16], wurde der Fragebogen um einen weiteren Teil zum Umgang mit COVID-19-Patienten in Bezug auf die VTE-Prophylaxe und Antikoagulationstherapie ergänzt, welcher von 31 der insgesamt 67 Antwortenden bearbeitet wurde. Die Verfügbarkeit der Testmöglichkeiten unterschied sich dabei nicht wesentlich vom Gesamtkollektiv (Zusatzmaterial online: Zusammenstellung – Abb. 2). Auf 25,8 % der teilnehmenden Intensivstationen erhielten COVID-19-Patienten eine andere Art der VTE-Prophylaxe als andere Patienten mit Sepsis (Tab. 3).

**Table 3:** 

	*n*	%
*Führen Sie bei zuvor gesunden Patienten mit COVID-19 generell eine andere medikamentöse VTE-Prophylaxe bzw. Antikoagulation durch als bei in der Krankheitsschwere vergleichbaren Patienten mit Sepsis?*		
Ja	8	25,8
Nein	23	74,2
K. A.	0	0
*Wann beginnen Sie mit der Antikoagulation bei COVID-19-Patienten?*		
Prophylaktisch auf der Normalstation	22	71,0
Prophylaktisch bei Aufnahme auf der ITS	5	16,1
Auf NST bei erhöhten D‑Dimeren oder Nachweis von Thrombose	3	9,7
Auf ITS bei erhöhten D‑Dimeren oder Nachweis von Thrombose	1	3,2
*Screenen Sie Patienten mit COVID-19 mithilfe eines Scores auf das Vorliegen einer Koagulopathie?*		
Ja	5	16,1
Nein	26	83,9
*Falls „ja“, mit welchem Score?*		
JAAM	2	6,5
ISTH	2	6,5
Anderer Score	1	3,2
*Falls „nein“, screenen Sie mittels D‑Dimeren?*		
Nur mit D‑Dimeren	9	29,0
Kein Screening	17	54,8

*ITS* Intensivstation, *ISTH* International Society of Thrombosis and HemostasisInternational Society on Thrombosis and Haemostasis, *JAAM* Japanese Association for Acute Medicine, *NST* Normalstation, *VTE* venöse Thrombo-Embolie

Gezielt nach der Strategie zur VTE-Prophylaxe bzw. Antikoagulation bei COVID-19-Patienten gefragt, gaben die meisten teilnehmenden ITS an, eine VTE-Prophylaxe mit NMH ohne Anti-FXa-Aktivitätsbestimmung (41,9 %) durchzuführen. Danach wurden in absteigender Häufigkeit die Gabe von UFH (nicht PTT-wirksam, 35,5 %) und NMH mit Anti-FXa-Aktivitätsbestimmung (19,4 %; Zusatzmaterial online: Zusammenstellung – Tab. 3; Abb. 2b) genannt. In Häusern, in denen für COVID-19-Patienten ein anderes Konzept für die Antikoagulation verfolgt wird als bei anderen Patienten mit Sepsis, wird UFH deutlich häufiger PTT-wirksam eingesetzt, 25,0 % vs. 9,4 %. Auch Argatroban findet häufiger Verwendung.

## Diskussion

Die Deutsche Sepsis Gesellschaft (DSG) empfiehlt in Ihrer „S3-Leitlinie Sepsis – Prävention, Diagnostik, Therapie und Nachsorge“ [1] in Übereinstimmung mit den Leitlinien der Surviving Sepsis Campaign [20], der European Society of Anesthesiology and Intensive Care (ESAIC) [3] und der Deutschen Gesellschaft für Angiologie [2] allgemein eine „pharmakologische Prophylaxe einer venösen Thromboembolie (VTE) mittels unfraktioniertem Heparin (UFH) oder niedermolekularem Heparin (NMH), sofern keine Kontraindikationen in Bezug auf die Verwendung dieser Wirkstoffe vorliegen“. Aufgrund der heterogenen Datenlage wird explizit keine Empfehlung bezüglich der Bevorzugung von einem beiden Medikamente oder dem Applikationsweg (i.v. vs. s.c.) abgeben. Auch werden weder eine Dosierung für eine der genannten Substanzen, noch ein (laborchemisches) Zielkriterium für die antikoagulatorische Wirkung genannt. Die vorliegende Umfrage zeigt, dass diese aktuellen Leitlinienempfehlungen auf deutschen Intensivstationen in der klinischen Praxis sehr heterogen interpretiert und umgesetzt werden. Die meisten Kliniken setzen bei Patienten mit Sepsis (ohne Schock) UFH oder NMH in relativ niedrigen Dosierungen ein, ohne dabei ein spezifisches Gerinnungsmonitoring durchzuführen. Dies ist leitliniengerecht, bei Patienten im septischen Schock sollte jedoch das Risiko von Unterdosierungen bei katecholaminpflichtigen Patienten beachtet werden. So schlussfolgern Minet et al. nach einer Auswertung der verfügbaren Literatur [17], dass eine Kontrolle des Anti-FXa-Spiegels bei diesen Patienten insofern ratsam sei, als dass Katecholamine mit der Aufnahme von s.c.-applizierten Substanzen interagieren, jedoch die Veränderung der Anti-FXa-Wirkung nicht direkt mit der Katecholamindosis korreliert [17, 19]. Obwohl bei Patienten mit einer sekundären Peritonitis nach operativer Sanierung (ohne Schock) UFH etwas häufiger eingesetzt wird, kommen auch hier beide Varianten regelhaft zum Einsatz. Einzelne Kliniken führen eine medikamentöse VTE-Prophylaxe mit PTT-Zielwerten > 40 s durch. Die Bestimmungen der PTT sowie der ACT zum Therapiemonitoring von Heparin bei Akute-Phase-Reaktionen wie in der Sepsis sind jedoch aus mehreren Gründen mit Unsicherheiten verbunden: So können zum einen die Werte z. B. durch Verlust wie auch durch Hochregulation einzelner Faktoren in der Akute-Phase-Reaktion beeinflusst werden [24], zum anderen unterliegt die Messung von Gerinnungsparametern wie der PTT einer hohen Variabilität zwischen einzelnen Laboren und Institutionen, wodurch die vergleichende Bewertung erschwert ist [18].

Auch Argatroban kommt zum Einsatz, obwohl die Zulassung des Medikamentes die Indikation zur VTE-Prophylaxe formal nicht beinhaltet. Interessant ist, dass der Einsatz von mechanischen Verfahren zur VTE-Prophylaxe nur in weniger als 10 % der Fälle angegeben wurde. Dies steht im Gegensatz zu den entsprechenden Leitlinienempfehlungen, die „eine Kombination aus einer pharmakologischen VTE-Prophylaxe und einer mechanischen Prophylaxe [vorschlagen], wann immer dies möglich ist“ [1, 20]. Zudem scheint die Datenlage zumindest für die IPK solide [14]. Allerdings wurde auch die entgegengesetzte Angabe „keine mechanische Kompression“ nur in 10–15 % der Antworten gemacht, sodass definitive Aussagen hierzu nicht möglich sind.

Da die SIC eine schwerwiegende Komplikation einer Sepsis ist [7, 26], liegt es nahe zu überlegen, ob Sepsispatienten oder zumindest solche, die Anzeichen für eine beginnende SIC ohne Blutungszeichen haben, von einer, über die VTE-Prophylaxe hinausgehenden, Antikoagulation profitieren könnten [12]. Jedoch fehlt die Evidenz, wann und mit welchem Medikament eine solche Therapie indiziert ist. In den bisher vorliegenden Studien gelang es mit keinem der getesteten Therapieregime, die Überlebenswahrscheinlichkeit eines unselektierten septischen Patientenkollektivs zu erhöhen. Anders könnte dies in den Subgruppen von Patienten mit SIC oder schwerer Sepsis ohne manifeste Blutungskomplikationen aussehen, wie eine retrospektive, multizentrische Kohortenstudie [30] sowie 2 kürzlich publizierte systematische Übersichtsarbeiten mit Metaanalyse [27, 29] suggerieren. Yamakawa et al. konnten das Subkollektiv sogar noch weiter eingrenzen und zeigen, dass insbesondere diejenigen Patienten von einer „erweiterten“ Antikoagulation profitieren, die eine nachgewiesene SIC und/oder eine hohe, jedoch keine sehr hohe (initialer Sequential Sepsis-related Organ Failure Assessment (SOFA) Score 13–17) Erkrankungsschwere aufweisen [28]. Unsere Umfrage zeigt, dass auf immerhin 20 % der deutschen Intensivstationen bereits bei Anzeichen für eine SIC eine Antikoagulation durchgeführt wird, die über die reine medikamentöse VTE-Prophylaxe hinausgeht. Bemerkenswert ist dabei, dass diese Antikoagulation auf immerhin 10 % der ITS auch dann noch bei Patienten mit PS und SIC fortgeführt wird, wenn es zum Auftreten einer diffusen Blutung kommt. Bei Patienten mit abdomineller Sepsis, SIC und diffuser Blutung ist dies nicht der Fall; nur eine der teilnehmenden Kliniken führt in dieser Situation noch eine PTT-wirksame Antikoagulation durch.

Obwohl sich die Anzeichen und Berichte aus aller Welt mehren, dass insbesondere schwere Verläufe von COVID-19 mit einer Koagulopathie vergesellschaftet sind [9, 16, 21], wurde nur in etwa 25 % der in der vorliegenden Umfrage repräsentierten Intensivbereiche eine Anpassung der VTE-Prophylaxe vorgenommen. In den Kliniken, die im Rahmen der COVID-19-Pandemie neue Schemata einführten, wird UFH häufiger PTT-wirksam eingesetzt, und es kommt deutlich häufiger Argatroban zur Anwendung. Ein gezieltes Screening auf eine SIC mithilfe eines Scores findet auch in dieser Risikogruppe nur in 5 % der Kliniken statt. Auch eine regelmäßige laborchemische Bestimmung von D‑Dimeren wird in nur knapp einem Drittel der Kliniken durchgeführt, sodass geschlussfolgert werden kann, dass die koagulatorischen Besonderheiten bei COVID-19-Patienten in den meisten Häusern nur im Falle von manifesten, symptomatischen Komplikationen (z. B. Thrombose, Embolie etc.) behandelt werden. Dies deckt sich nur z. T. mit den Empfehlungen der European Society of Cardiology, die zuletzt im Juni 2020 aktualisiert wurden und die bei COVID-19-Patienten eine „normale“ VTE-Prophylaxe empfehlen, jedoch die Hinweise geben, dass ein erhöhtes Risiko für Lungenarterienembolien besteht, und dass D‑Dimere daher regelmäßig kontrolliert werden sollen [25]. Allerdings gehen auch die Empfehlungen der unterschiedlichen Fachgesellschaften zur Antikoagulation bei COVID-19 derzeit noch z. T. weit auseinander [5]. So wird beispielsweise in der Ende November 2020 veröffentlichten interdisziplinären S2k-Leitlinie zur stationären Behandlung von COVID-19-Patienten für intensivmedizinisch versorgte Patienten, eine „intensivierte“ VTE-Prophylaxe mit „halbtherapeutischem“ NMH oder UFH mit einem Ziel-PTT-Bereich von 1,5–1,8 s empfohlen [15].

Limitierend bei der Bewertung unserer Daten sind die Charakteristika einer anonymen Umfrage sowie der Umstand, dass, vor dem Hintergrund einer Gesamtzahl von über 1000 Intensivstationen in Deutschland, eine Repräsentativität unserer Umfrageergebnisse nicht belegt werden kann; diese sollte jedoch angesichts der breiten Streuung der Umfrage und der hohen relativen Anzahl an teilnehmenden Universitätsklinika zumindest in gewissem Maße angenommen werden können.

### Zusammenfassung

Aufgrund der ausgesprochenen Heterogenität der Ergebnisse muss zusammenfassend konstatiert werden, dass weiterhin relevante Unklarheiten in Bezug auf den Stellenwert der VTE-Prophylaxe sowie den möglichen Benefit einer therapeutischen Antikoagulation bei Sepsis bestehen. Um dem Anspruch einer evidenzbasierten Medizin auch in diesem Feld der Intensivtherapie gerecht werden zu können, bedarf es daher einer systematischen Bearbeitung dieses Themenfeldes. Ziel muss es sein, klinische Studien auf den Weg zu bringen, die es zulassen, sowohl die medikamentöse VTE-Prophylaxe als auch eine therapeutische Antikoagulation evidenzbasiert patientenindividuell oder zumindest subgruppengerecht anzuwenden.

## Supplementary Information





