# Supplementary material for: Treatment of sepsis-induced coagulopathy: Results of a Germany-wide survey in intensive care units
Source: Anaesthesist. 2021 Feb 8;70(8):662–70. [Article in German] doi: 10.1007/s00101-021-00916-9 (PMC7869761; doi:10.1007/s00101-021-00916-9)
Supplement: Supplementary file 1 [file 101_2021_916_MOESM1_ESM.pdf]

Zusatzmaterial zum Beitrag „Therapie der Sepsis-induzierten Koagulopathie. Ergebnisse einer deutschlandweiten Umfrage auf Intensivstationen“ von Schmoch T, Brenner T, Becker-Pennrich A et al. (2021) in *Der Anaesthesist.*

Beitrag und Zusatzmaterial stehen Ihnen auf www.springermedizin.de zur Verfügung. Bitte geben Sie dort den Beitragstitel in die Suche ein.

Tabelle 1 – Scores zur Testung auf Disseminierte Koagulopathie

	ISTH – Score (12)	JAAM – Score (11)	SIC – Score (3,4)	Punkte
Grunderkrankung, die eine DIC wahrscheinlich macht	erforderlich	erforderlich	Nicht erforderlich	
SIRS score	-	0-2		0
		≥3		1
Thrombozytenzahl	>100/nL	≥120/nL	>150	0
	≤100/nL	80-120/nL (oder Abfall um >30% /24 h)	>100 und <150/nL	1
	≤50/nL			2
		<80/nL (oder Abfall um >50% /24 h)		3
Prothrombin time (PT-Ratio)	<3 sec	(<1.2)		0
	3-6 sec	(≥1.2)	(≥1,2 und <1,4)	1
	≥6 sec		(≥1,4)	2
Fibrinogen	>100 mg/dL	-	-	0
	-	-	-	1
	≤100 mg/dL	-	-	2
FDP/D-Dimere	Nicht erhöht	<10 µg/mL	-	0
		10-25 µg/mL	-	1
	leicht erhöht		-	2
	deutlich erhöht	≥25 µg/mL	-	3
SOFA-Score			1	1
			≥ 2	2
Total DIC score	<5 Punkte	<4 Punkte	<4 Punkte	Keine DIC/SIC
	≥5 Punkte	≥4 Punkte	≥4 Punkte	DIC/SIC

ISTH International Society on Thrombosis and Haemostasis, *JAAM* Japanese Association for Acute Medicine, *DIC* disseminated intravascular coagulation, *SIRS* systemic inflammatory response syndrome, SIC Sepsis Induced Coagulopathy, SOFA Sequential (Sepsis-related) Organ Failure Assessment Score;

Fallbeispiel 1 – zur pneumogenen Sepsis mit Variationen.

Nicht operativer Patient, 68 Jahre, Pneumonie, intubiert, beatmet mit FiO2 0,60, kein bis niedriger Katecholaminbedarf (kein weiteres Organversagen, kein Schock) und zusätzlich:

a) keine weiteren Erkrankungen.
b) HIT Typ II in der Anamnese, Jahre zurückliegend, aktuell kein Hinweis auf thromboembolische Ereignisse oder Thrombozytenabfall.
c) akute HIT Typ II, Thrombozytenabfall, Thrombosen, HIT-Schnelltest positiv.
d) orale Antikoagulation mit DOAK bei vorbestehendem chronischem Vorhofflimmern. (Mehrfachangaben sind möglich)
e) orale Antikoagulation mit Marcumar bei vorbestehendem chronischem Vorhofflimmern.
f) vorbestehend orale Antikoagulation mit DOAK bei Z.n. mehrfachen TVT und LE vor Jahren.
g) vorbestehend orale Antikoagulation mit Marcumar bei Z.n. mehrfachen TVT und LE vor Jahren.
h) Sepsis mit Nachweis von bakteriellen Erregern in der Blutkultur, mittlerer bis hoher Katecholaminbedarf UND DIC (livide Akren, Thrombozytenzahl 40.000 G/l, Quick < 50 %, D-Dimere stark erhöht, Fibrinogen < 100 mg/dl), aber keine Blutungssymptomatik.
i) Sepsis mit Nachweis von bakteriellen Erregern in der Blutkultur, mittlerer bis hoher Katecholaminbedarf besteht UND DIC (livide Akren, Thrombozytenzahl 40.000 G/l, Quick < 50 %, D-Dimere stark erhöht, Fibrinogen < 100 mg/dl), massive diffuse Blutungsneigung.

Fallbeispiel 2 – zur abdominellen Sepsis mit Variationen.

Operativer Patient, 68 Jahre, keine kardiovaskulären Vorerkrankungen, abdominelle Sepsis, beatmet mit FiO2 0,45, kein bis niedriger Katecholaminbedarf (kein weiteres Organversagen, kein Schock), nach ausgedehnter fokussanierender abdomineller Operation, mit Blutverlust von ca. 1,5 l und stattgehabter Transfusion von allogenen Blutprodukten, Drainagen einliegend, aktuell keine aktive Blutung:

a) am 2. postoperativen Tag.
b) am 2. postoperativen Tag aber DIC, livide Akren, Thrombozytenzahl 40.000 G/l, Quick < 50 %, D-Dimere stark erhöht, Fibrinogen < 100 mg/dl, mittlerer bis hoher Katecholaminbedarf, keine Blutung.
c) am 2. postoperativen Tag aber DIC, livide Akren, Thrombozytenzahl 40.000 G/l, Quick < 50 %, D-Dimere stark erhöht, Fibrinogen < 100 mg/dl, mittlerer bis hoher Katecholaminbedarf, massive diffuse Blutungsneigung.
d) am 2. postoperativen Tag, HIT Typ II in der Anamnese, Jahre zurückliegend, aktuell kein Hinweis auf thromboembolische Ereignisse oder Thrombozytenabfall.
e) am 2. postoperativen Tag bei akuter HIT Typ II, Thrombozytenabfall, Thrombosen, HIT-Schnelltest positiv.
f) am 2. postoperativen Tag bei DOAK als Vormedikation.
g) am 2. postoperativen Tag bei Marcumar als Vormedikation.

Ausführlicher Methodenteil

Grundlage unserer Erhebung war eine Deutschland-weite Online-Umfrage zwischen Oktober 2019 und April 2020. Der Fragebogen richtete sich gezielt an die **ärztlichen Leiter einer Intensivstation (ITS)** oder eines Intensivbereiches mit dem explizit formulierten Ziel, nur einen ausgefüllten Fragebogen pro Intensivbereich zu erhalten. Eine doppelte Teilnahme war allerdings technisch nicht blockiert. Der webbasierte Fragebogen (**Online Zusatzmaterial Fragebogen**) wurde von der der Arbeitsgruppe perioperative biomedizinische Informatik der Klinik für Anaesthesiologe des Klinikums der Universität München programmiert und über das System „Django-Survey" aufgesetzt. Der Link zu dem Fragebogen wurde zunächst im Oktober und November 2019 an die teilnehmenden Kliniken der SepNet-Studiengruppe verschickt und über diese an weitere Krankenhäuser unterschiedlicher Versorgungsstufen weitergeleitet. In einem zweiten Schritt wurden im April 2020 alle in der Deutschen Interdisziplinären Vereinigung für Intensiv- und Notfallmedizin (DIVI) organisierten intensiv-medizinischen Abteilungen per eMail zur Teilnahme an der Umfrage eingeladen. Am 14.05.2020 wurde die Umfrage zudem noch über den Verteiler der IAKH (Interdisziplinäre Arbeitsgemeinschaft für Klinische Hämotherapie) verschickt. Die Umfrage wurde schließlich am 31.05.2020 geschlossen. Die Teilnahme erfolgte vollständig anonymisiert. Es wurden keine personenbezogenen Daten erhoben.

Der Online-Fragebogen wurde nach ausführlicher Literaturrecherche und in enger Rücksprache mit den ausgewiesenen Experten der SepNet Study Group erstellt. Einfach- und Mehrfach-Auswahlfragen sowie Freitextfelder wurden zur Erhebung genutzt. Er umfasste insgesamt fünf Fragenkomplexe zu (1.) **„Infrastruktur"**, (2.) **„Status quo Antikoagulation"**, (3.) **„Status quo Sepsis**, (4.) „**Status quo Sepsis assoziierte DIC**" und (5.) "**COVID-19**". Kern des Fragebogens waren zwei Fallvignetten von Patienten mit pneumogener (**Online Zusatzmaterial Zusammenstellung, Fallbeispiel 1** bzw. abdomineller Sepsis bei sekundärer Peritonitis und erfolgter chirurgischer Fokussanierung (**Online Zusatzmaterial Zusammenstellung, Fallbeispiel 2**). Nachfolgend wurden leichte Variationen der Fälle (a-i für Fall 1 sowie a-g für Fall 2) vorgenommen und die Teilnehmer der Umfrage gebeten anzugeben, wie sie den entsprechenden Patienten nun in der jeweiligen Situation antikoagulatorisch behandeln würden. In die hier vorliegende Auswertung

wurden nur die Fallvariationen einbezogen, die sich mit einer Sepsis ohne weitere Komplikationen, einer Sepsis mit einer leichten SIC sowie einer Sepsis mit SIC und diffuser Blutung befassten (a, h und i für Fall 1 sowie a-c für Fall 2). Neben auswählbaren Antwortmöglichkeiten gab es Freitextfelder und die Teilnehmer waren gebeten, die verwendeten Medikamente und die in ihrem Intensivbereich gängigen Dosierungen für einem der Fallvignette entsprechenden Patienten anzugeben.

Der letzte Teil des Fragebogens, der sich mit den Besonderheiten bei COVID-19-Patienten befasst, wurde im April 2020 online gestellt und war zwischen dem 01.04.2020 und dem 31.05.2020 online. Somit konnte nur ein Teil der Teilnehmer die Fragen aus diesem Teil beantworten.

Statistik

Zur statistischen Auswertung wurden deskriptive Methoden mittels Microsoft® Office Excel (Excel für Mac Version 16.3, Microsoft Corporation, Redmond, Washington, Vereinigte Staaten) und Prism® 8 for macOS, (GraphPad Prism® für Mac Version 8.3.0; GraphPad Software LLC, San Diego, Vereinigte Staaten) verwendet.Es wurden absolute und relative Häufigkeiten dargestellt. Das arrhythmische Mittel, Median und Quartile wurden berechnet, wenn immer es sinnvoll erschien.

Abbildung 1 – Verfügbarkeit von viskoelastischem und aggregometrischem Gerinnungsmonitoring nach Klinikart.

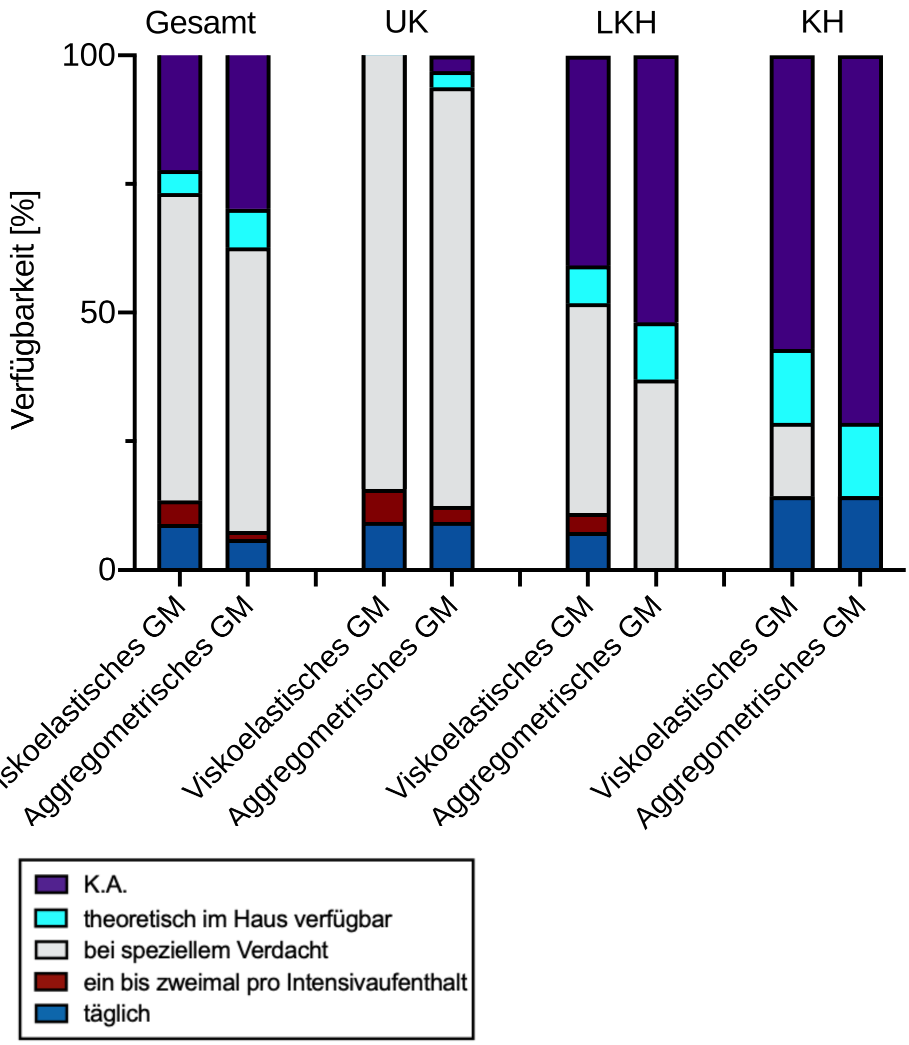

GM = Gerinnungsmonitoring; UK = Universitätsklinika (n = 32); LKH = akademische Lehrkrankenhäuser (n = 27); KH = Krankenhäuser (n = 7).

Tabelle 2 – Tabellarische Auflistung der Antworten (absolute und relative Häufigkeiten) zu den Fallvignetten.

	pneumogene Sepsis		abdominielle Sepsis		pneumogene Sepsis + SIC		pneumogene Sepsis + SIC + Blutung		abdominielle Sepsis + SIC		abdominielle Sepsis + SIC + Blutung	
	n	%	n	%	n	%	n	%	n	%	n	%
Anzahl der abgegebenen Antworten	67		66		65		64		67		64	
Anzahl der Antworten incl. Mehrfachnennungen	102		91		81		88		87		80	
keine Antikoagulation	1	1,5%	1	1,5%	3	4,6%	40	62,5%	7	10,4%	45	70,3%
UFH nicht PPT wirksam	23	34,3%	34	51,5%	41	63,1%	17	26,6%	41	61,2%	17	26,6%
UFH + PPT wirksam	2	3,0%	2	3,0%	13	20,0%	6	9,4%	14	20,9%	1	1,6%
NMH ohne Xa Bestimmung	43	64,2%	29	43,9%	4	6,2%	0	0,0%	3	4,5%	1	1,6%
NMH + Xa Bestimmung	8	11,9%	7	10,6%	4	6,2%	3	4,7%	4	6,0%	0	0,0%
Fondaparinux	1	1,5%	0	0,0%	0	0,0%	0	0,0%	0	0,0%	0	0,0%
Argatroban	1	1,5%	1	1,5%	4	6,2%	1	1,6%	3	4,5%	1	1,6%
Danaparoid	0	0,0%	0	0,0%	0	0,0%	0	0,0%	0	0,0%	0	0,0%
Apixaban/Edoxaban od. Rivaroxaban	0	0,0%	0	0,0%	0	0,0%	0	0,0%	0	0,0%	0	0,0%
Keine mechanische Kompression	6	9,0%	4	6,1%	5	7,7%	6	9,4%	6	9,0%	3	4,7%
MTPS	10	14,9%	9	13,6%	5	7,7%	9	14,1%	4	6,0%	7	10,9%
IPK	6	9,0%	4	6,1%	2	3,1%	5	7,8%	5	7,5%	5	7,8%
MTPS+IPK	1	1,5%	0	0,0%	0	0,0%	1	1,6%	0	0,0%	0	0,0%

Freitext 1 – zur VTE-Prophylaxe bei pneumogener und abdomineller Sepsis.

Mehrere Teilnehmer nutzten die Möglichkeit zur Eingabe freier Kommentare.

Für den Fall einer **pneumogenen Sepsis ohne weitere Komplikationen** gaben 33 der 67 Antwortenden (49,3%) Freitextkommentare ab und spezifizierten damit ihre Angaben. 19-mal wurde Enxoaparin als verwendetes Medikament genannt (17 x 0,4ml/d (= 40mg = 4000 I.E.); 1 x „nach KG"; 1 x „0,4-0,6 mit Ziel Anit-Xa ca. 0,4). UFH kam fünfmal in den Freitextantworten vor, wobei dreimal PTT-Ziele zwischen 25 und 40s genannt wurden und einmal „200, 300 oder 500 I.E./h ohne PTT Ziel", sowie einmal „6 I.E./kg KG/h mit Ziel PTT 40s"

Für den Fall der **abdominellen Sepsis (bei sekundärer Peritonitis) ohne weitere Komplikationen,** wurden von 27 der 66 Antwortenden (40,9%) Freitextkommentare abgegeben. 15-mal wurde Enoxaparin genannt. In 11 von 15 Antworten gaben die Teilnehmer an, standardmäßig 0,4ml zu verwenden, dreimal wurden 0,4-0,6ml genannt und einmal wurde „nach Gewicht" als Antwort gegeben, ohne dies genauer zu spezifizieren. Heparin intravenös i.v. wurde 5-mal angegeben, wobei Zielwerte für die PTT von 25-40s bzw. in einem Fall bis 45s angegeben wurden. In einer Antwort wurde angegeben, dass Argatroban bei Patienten mit abdomineller Sepsis ab dem 3. postoperativen Tag als medikamentöse Standard-VTE-Prophylaxe verwendet wird. Eine Dosis oder eine Ziel-ACT oder PTT wurden hierfür nicht genannt.

In beiden Fällen (PS und AS) wurde viermal Certoparin 3000 I.E. pro Tag genannt und Tinzaparin (4500 I.E./d) und Nardoparin (3800 I.E. <70kg KG und 5700 I.E. >70kg) jeweils einmal.

Freitext 2 – zur VTE-Prophyalxe und Antikoagulation bei PS mit SIC mit und ohne Blutungsneigung.

Für den Fall einer **pneumogenen Sepsis mit SIC aber ohne Blutung** gaben 26 der 65 Antwortenden (40,0%) Freitextkommentare ein. 19-mal wurde UFH als primär eingesetztes Therapeutikum genannt, wobei die Dosisschemata deutlich variierten. Sechsmal wurden Dosisangaben zwischen 100 IE/h und 500 I.E./h gemacht ohne, dass ein PTT-Ziel angegeben wurde. Einmal wurden 6 I.E./kgKG/h genannt und dreimal PTT-Zielwerte von <40s. In einer Antwort wurden 500-800 I.E./h angegeben, um ein PTT-Ziel von 35s zu erreichen. In zwei Antworten wurde ein PTT-Ziel von 40-50s genannt, in zwei 50-60s und in einer sogar 60-70s. Eine Antwort enthielt „PTT-

gesteuert“ ohne eine konkrete Sekundenangabe und eine „low-dose“ ohne weitere Spezifikation. Die Gabe Argatroban mit einer Ziel-PTT von 40-60s kam einmal vor, ebenso Enoxaparin „40-60mg/d i.v.“ und Enoxaparin „2 x 1mg/kgKG mit Ziel-Anti-Xa 0,4-0,8“. Ebenfalls jeweils einmal kamen die Gabe von AT und die eventuelle Substitution von Ceprotin (nach Protein C Messung) oder gefrorenen Frischplasmen (GFPs) vor.

17 der 64 Antwortenden (26,6%) im Fall einer **pneumogenen Sepsis mit SIC und diffuser Blutungsneigung** gaben Freitextkommentare. Dabei wurden viermal die Gabe von AT und jeweils zweimal die Substitution von GFPs, Fibrinogen sowie der Einsatz von Tranexamsäure genannt. Dreimal wurde angegeben, dass die Antikoagulation pausiert würde, und dreimal wurde UFH mit PTT-Zielwerten < 40s angegeben bzw. einmal einfach „low dose“ in das Freitextfeld eingetragen. Eine der antwortenden Kliniken gab an, 2-3x 5000 I.E. s.c. zu spritzen. Eine Antwort unterschied sich deutlich von allen anderen, da in ihr ein PTT-Zielbereich von 60-70s definiert wurde.

Freitext 3 – zur VTE-Prophylaxe und Antikoagulation bei PS und AS mit SIC mit und ohne Blutungsneigung.

Für den Fall einer **abdomniellen Sepsis mit SIC aber ohne Blutung** gaben 23 der 67 Antwortenden (34,0%) Freitextkommentare ein. In 18 dieser Antworten wurde UFH genannt. Viermal wurden dabei PTT-Ziele <45s und dreimal zwischen 40 und 50s angegeben. Auf einer ITS liegt das PTT-Ziel in diesem Fall bei 60-70s. Eine ITS würde die Antikoagulation pausieren. Zwei geben als einziges Ziel eine AT-Aktivität von >70% an. Es werden mehrfach Heparindosierungen von 100-400 I.E./h oder 6 I.E./kg KG/h genannt. Zweimal wird Enoxaparin 40-60mg erwähnt.

Für den Fall einer **abdomniellen Sepsis mit SIC und diffuser Blutungsneigung** gaben 13 der 64 Antwortenden (20,3%) Freitextkommentare ein. Dabei wurde AT-Substitution (3x) und AT<70% (1x) genannt. Eine teilnehmende ITS würde die Antikoagulation pausieren, dreimal wird die Fibrinogen-Substitution genannt, zweimal die Gabe von GFPs und Tranexamsäure (2x). UFH wird in 6 Freitextantworten mit Dosierungen zwischen 100 und 1000 I.E. mit Zielangaben von „low-dose“ bis PTT 60-70s genannt.

Abbildung 2 – Verfügbarkeit von Laborwerten in den Kliniken, die an dem Umfrageteil zur VTE-Prophylaxe und Antikoagulation bei COVID-19 teilgenommen haben.

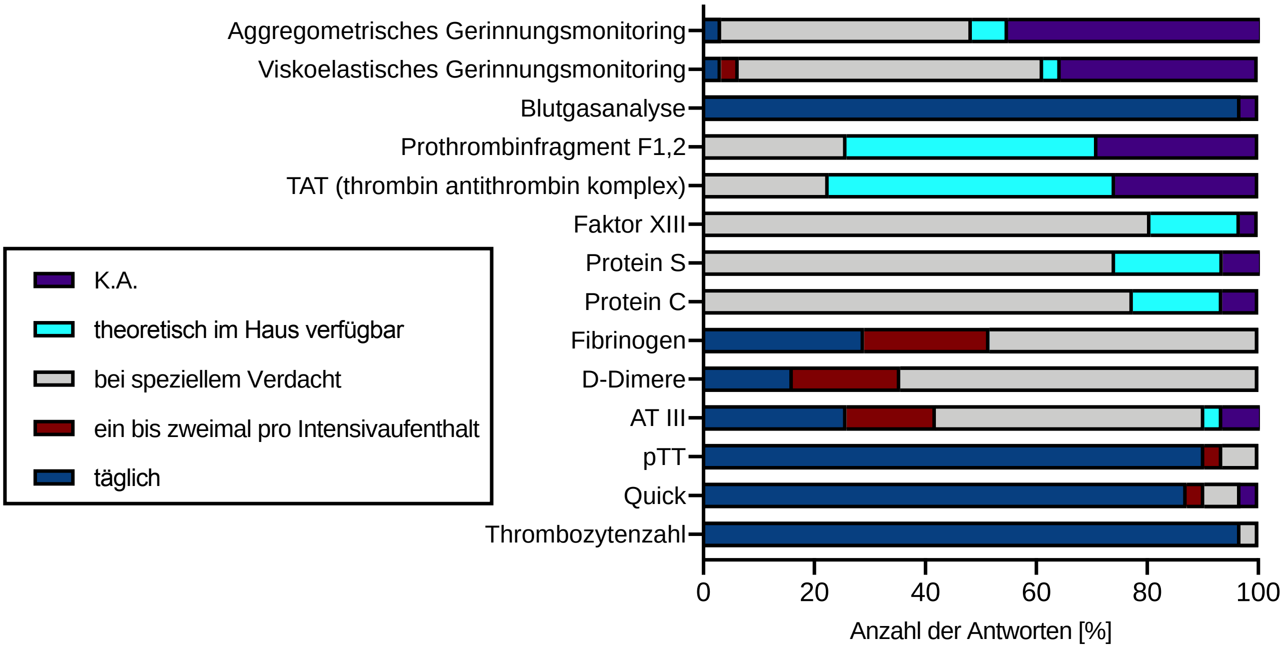

Tabelle 3 – Antikoagulationskonzepte bei COVID-19.

	COVID-19 gesamt		andere Antikoagulation bei COVID-19 als bei Sepsis allgemein		keine andere Antikoagulation bei COVID-19 als bei Sepsis allgemein	
	n	%	n	%	n	%
Anzahl der abgegebenen Antworten	31		8		23	
Anzahl der Antworten incl. Mehrfachnennungen	39		12		27	
keine Antikoagulation	0	0,0%	0	0,0%	0	0,0%
UFH nicht PPT wirksam	11	35,5%	2	25,0%	9	39,1%
UFH + PPT wirksam	3	9,7%	2	25,0%	1	4,3%
NMH ohne Xa Bestimmung	13	41,9%	3	37,5%	10	43,5%
NMH + Xa Bestimmung	6	19,4%	2	25,0%	4	17,4%
Fondaparinux	0	0,0%	0	0,0%	0	0,0%
Argatroban	3	9,7%	2	25,0%	1	4,3%
Danaparoid	0	0,0%	0	0,0%	0	0,0%
Apixaban/Edoxaban od. Rivaroxaban	0	0,0%	0	0,0%	0	0,0%
Keine mechanische Kompression	1	3,2%	0	0,0%	1	4,3%
MTPS	2	6,5%	1	12,5%	1	4,3%
IPK	0	0,0%	0	0,0%	0	0,0%
MTPS+IPK	0	0,0%	0	0,0%	0	0,0%
