# Supplementary material for: Treatment of sepsis-induced coagulopathy: Results of a Germany-wide survey in intensive care units
Source: Anaesthesist. 2021 Feb 8;70(8):662–70. [Article in German] doi: 10.1007/s00101-021-00916-9 (PMC7869761; doi:10.1007/s00101-021-00916-9)
Supplement: Supplementary file 2 [file 101_2021_916_MOESM2_ESM.pdf]

ATISS-DIC

Sehr geehrte Frau Kollegin, sehr geehrter Herr Kollege, zunächst einmal möchten wir uns ganz herzlich dafür bedanken, dass Sie sich die Zeit für die Teilnahme an unserer Umfrage nehmen, die sich mit dem Status quo der Antikoagulation auf Intensivstation und der Erkennung bzw. Behandlung von Patienten mit Sepsis-assoziierter Disseminierter Intravasaler Coagulopathie (DIC) befasst. Unsere bereits letztes Jahr begonnene Umfrage hat durch die COVID-19 Pandemie aktuell stark an Aktualität gewonnen, diesem Umstand wird mit einem eigenen Fragenblock zum Thema COVID-19 diesem Thema Rechnung getragen. Diese Umfrage ist an alle Kolleginnen und Kollegen gerichtet, die in Leitungsfunktion auf einer Intensivstation tätig sind. Die Bearbeitung des Fragebogens nimmt etwa 15 Minuten Ihrer Zeit in Anspruch. Um die auf Ihrer ICU jeweils zur Anwendung gebrachten antikoagulatorischen Konzepte bei verschiedenen Erkrankungsbildern bestmöglich erfassen zu können, haben wir Standardfallvignetten erstellt. Wir möchten Sie um Ihre Einschätzung bitten, wie ein solcher Patient auf Ihrer ITS antikoaguliert werden würde. Wenn eine Patientengruppe, z.B. abdominalchirurigsche Patienten, bei Ihnen nicht behandelt wird, klicken Sie auf „nicht zutreffend“. Nachdem für die dargestellten Situationen zwar durchaus Evidenzen für das ein oder andere Therapiekonzept existieren, jedoch für die meisten Fälle keine eindeutigen Vorgaben durch z.B. eine spezifische Leitlinie bestehen, gibt es für die meisten dargestellten Situationen keine „richtigen“ oder „falschen“ Antworten; die Intention der Umfrage ist die aktuelle Situation bzw. die gelebte Realität in Deutschland in dieser Thematik abzubilden.

Angaben zur Infrastruktur

Infrastruktur

Diesen Teil beantworten

Welchem Typ gehört Ihr Krankenhaus an?

○ **Krankenhaus** ○ **Lehrkrankenhaus** ○ **Universitätsklinik**

Über wie viele Betten verfügt Ihr Krankenhaus insgesamt?

○ **<100** ○ **100-250** ○ **251-500** ○ **501-1000** ○ **>1000**

Welcher Fachdisziplin obliegt die Führung Ihrer Intensivstation?

○ **Anästhesie** ○ **Allgemein- & Viszeralchirurgie** ○ **Neurochirurgie**
○ **Herzchirurgie** ○ **Pulmonologie** ○ **Kardiologie**
○ **Gastroenterologie- & Hepatologie** ○ **Hämatoonkologie** ○ **Pädiatrie**
○ **Neurologie** ○ **Sonstiges (bitte Freitext in nächster Frage)**

Welcher Fachdisziplin obliegt die Führung Ihrer Intensivstation? (Freitext)

[ ]

Patienten welcher Fachdisziplinen werden auf Ihrer Station behandelt?

- ☐ **Allgemein- & Viszeralchirurgie**
- ☐ **Traumatologie/Orthopädie**
- ☐ **Gefäßchirurgie**
- ☐ **Gynäkologie**
- ☐ **Pulmonologie**
- ☐ **Gastroenterologie- & Hepatologie**
- ☐ **Pädiatrie**
- ☐ **Sonstiges (bitte Freitext in nächster Frage)**
- ☐ **Neurochirurgie**
- ☐ **Herzchirurgie**
- ☐ **Thoraxchirurgie**
- ☐ **Urologie**
- ☐ **Kardiologie**
- ☐ **Hämatoonkologie**
- ☐ **Neurologie**

Patienten welcher Fachdisziplinen werden auf Ihrer Station behandelt? (Freitext)

Für wie viele Intensivbetten (nur Beatmungsbetten, keine IMC) ist Ihre Abteilung verantwortlich?

Wie hoch ist der durchschnittliche Anteil an invasiv beatmeten Patienten auf Ihrer Intensivstation? (Angabe in Prozent)

Wie ist die mittlere Verweildauer auf Ihrer Intensivstation? (Angabe in Tagen)

Status quo Antikoagulation

Status quo Antikoagulation

Diesen Teil beantworten

Gibt es eine Standard Operating Procedure (SOP) zum Thema der „Antikoagulation" auf Ihrer Intensivstation?

○ **ja** ○ **nein**

Fall 1. Nicht operativer Patient, 68 Jahre, Pneumonie, intubiert, beatmet mit FiO2 0,60, kein bis niedriger Katecholaminbedarf (kein weiteres Organversagen, kein Schock) und zusätzlich:
a) keine weiteren Erkrankungen.
(Mehrfachangaben sind möglich)

- ☐ **Keine Antikoagulation**
- ☐ **Unfraktioniertes Heparin (UFH) PTT wirksam**
- ☐ **Unfraktioniertes Heparin (UFH) nicht PPT wirksam**
- ☐ **Niedermolekulares Heparin (NMH) mit Anti-Xa-Bestimmung**
- ☐ **Niedermolekulares Heparin (NMH) ohne Anti-Xa-Bestimmung**
- ☐ **Argatroban**
- ☐ **Fondaparinux**
- ☐ **Danaparoid**
- ☐ **Keine mechanische Thromboseprophylaxe**
- ☐ **Intermittierende pneumatische Kompression (IPK)**
- ☐ **Medizinische Thromboseprophylaxestrümpfe (MTPS)**
- ☐ **nicht zutreffend**

zu Fall 1a): Bitte geben Sie, falls zutreffend, den Präparatenamen und die Dosierung bzw. den Präparatenamen und den Zielbereich an.

wie 1., jedoch zusätzlich:
b) HIT Typ II in der Anamnese, Jahre zurückliegend, aktuell kein Hinweis auf thromboembolische Ereignisse oder Thrombozytenabfall.
(Mehrfachangaben sind möglich)

- ☐ **Keine Antikoagulation**
- ☐ **Unfraktioniertes Heparin (UFH) PTT wirksam**
- ☐ **Unfraktioniertes Heparin (UFH) nicht PPT wirksam**
- ☐ **Niedermolekulares Heparin (NMH) mit Anti-Xa-Bestimmung**
- ☐ **Niedermolekulares Heparin (NMH) ohne Anti-Xa-Bestimmung**
- ☐ **Argatroban**
- ☐ **Fondaparinux**
- ☐ **Danaparoid**
- ☐ **Dabigatran**
- ☐ **Apixaban/Edoxaban oder Rivaroxaban**
- ☐ **Keine mechanische Thromboseprophylaxe**
- ☐ **Intermittierende pneumatische Kompression (IPK)**
- ☐ **Medizinische Thromboseprophylaxestrümpfe (MTPS)**
- ☐ **nicht zutreffend**

zu Fall 1b): Bitte geben Sie, falls zutreffend, den Präparatenamen und die Dosierung bzw. den Präparatenamen und den Zielbereich an.

wie 1., jedoch zusätzlich:
c) akute HIT Typ II, Thrombozytenabfall, Thrombosen, HIT-Schnelltest positiv.
(Mehrfachangaben sind möglich)

- ☐ **Keine Antikoagulation**
- ☐ **Unfraktioniertes Heparin (UFH) PTT wirksam**
- ☐ **Unfraktioniertes Heparin (UFH) nicht PPT wirksam**
- ☐ **Niedermolekulares Heparin (NMH) mit Anti-Xa-Bestimmung**
- ☐ **Niedermolekulares Heparin (NMH) ohne Anti-Xa-Bestimmung**
- ☐ **Argatroban**
- ☐ **Fondaparinux**
- ☐ **Danaparoid**
- ☐ **Dabigatran**
- ☐ **Apixaban/Edoxaban oder Rivaroxaban**
- ☐ **Keine mechanische Thromboseprophylaxe**
- ☐ **Intermittierende pneumatische Kompression (IPK)**
- ☐ **Medizinische Thromboseprophylaxestrümpfe (MTPS)**
- ☐ **nicht zutreffend**

zu Fall 1c): Bitte geben Sie, falls zutreffend, den Präparatenamen und die Dosierung bzw. den Präparatenamen und den Zielbereich an.

wie 1., jedoch zusätzlich:
d) orale Antikoagulation mit DOAK bei vorbestehendem chronischem Vorhofflimmern.
(Mehrfachangaben sind möglich)

- ☐ **Vorbestehende Antikoagulation absetzen**
- ☐ **nur vorbestehende Antikoagulation fortführen**
- ☐ **Unfraktioniertes Heparin (UFH) PTT wirksam**
- ☐ **Unfraktioniertes Heparin (UFH) nicht PPT wirksam**
- ☐ **Niedermolekulares Heparin (NMH) mit Anti-Xa-Bestimmung**
- ☐ **Niedermolekulares Heparin (NMH) ohne Anti-Xa-Bestimmung**
- ☐ **Argatroban**
- ☐ **Fondaparinux**
- ☐ **Danaparoid**
- ☐ **Dabigatran**
- ☐ **Apixaban/Edoxaban oder Rivaroxaban**
- ☐ **Marcumar**
- ☐ **Keine mechanische Thromboseprophylaxe**
- ☐ **Intermittierende pneumatische Kompression (IPK)**
- ☐ **Medizinische Thromboseprophylaxestrümpfe (MTPS)**
- ☐ **nicht zutreffend**

zu Fall 1d): Bitte geben Sie, falls zutreffend, den Präparatenamen und die Dosierung bzw. den Präparatenamen und den Zielbereich an.

wie 1., jedoch zusätzlich:
e) orale Antikoagulation mit Marcumar bei vorbestehendem chronischem Vorhofflimmern.
(Mehrfachangaben sind möglich)

- ☐ **Vorbestehende Antikoagulation absetzen**
- ☐ **nur vorbestehende Antikoagulation fortführen**
- ☐ **Unfraktioniertes Heparin (UFH) PTT wirksam**
- ☐ **Unfraktioniertes Heparin (UFH) nicht PPT wirksam**
- ☐ **Niedermolekulares Heparin (NMH) mit Anti-Xa-Bestimmung**
- ☐ **Niedermolekulares Heparin (NMH) ohne Anti-Xa-Bestimmung**
- ☐ **Argatroban**
- ☐ **Fondaparinux**
- ☐ **Danaparoid**
- ☐ **Dabigatran**
- ☐ **Apixaban/Edoxaban oder Rivaroxaban**
- ☐ **Marcumar**
- ☐ **Keine mechanische Thromboseprophylaxe**
- ☐ **Intermittierende pneumatische Kompression (IPK)**
- ☐ **Medizinische Thromboseprophylaxestrümpfe (MTPS)**
- ☐ **nicht zutreffend**

zu Fall 1e): Bitte geben Sie, falls zutreffend, den Präparatenamen und die Dosierung bzw. den Präparatenamen und den Zielbereich an.

wie 1., jedoch zusätzlich:
f) vorbestehend orale Antikoagulation mit DOAK bei Z.n. mehrfachen TVT und LE vor Jahren.
(Mehrfachangaben sind möglich)

- ☐ **Vorbestehende Antikoagulation absetzen**
- ☐ **nur vorbestehende Antikoagulation fortführen**
- ☐ **Unfraktioniertes Heparin (UFH) PTT wirksam**
- ☐ **Unfraktioniertes Heparin (UFH) nicht PPT wirksam**
- ☐ **Niedermolekulares Heparin (NMH) mit Anti-Xa-Bestimmung**
- ☐ **Niedermolekulares Heparin (NMH) ohne Anti-Xa-Bestimmung**
- ☐ **Argatroban**
- ☐ **Fondaparinux**
- ☐ **Danaparoid**
- ☐ **Dabigatran**
- ☐ **Apixaban/Edoxaban oder Rivaroxaban**
- ☐ **Marcumar**
- ☐ **Keine mechanische Thromboseprophylaxe**
- ☐ **Intermittierende pneumatische Kompression (IPK)**
- ☐ **Medizinische Thromboseprophylaxestrümpfe (MTPS)**
- ☐ **nicht zutreffend**

zu Fall 1f): Bitte geben Sie, falls zutreffend, den Präparatenamen und die Dosierung bzw. den Präparatenamen und den Zielbereich an.

wie 1., jedoch zusätzlich:
g) vorbestehend orale Antikoagulation mit Marcumar bei Z.n. mehrfachen TVT und LE vor Jahren.
(Mehrfachangaben sind möglich)

- ☐ **Vorbestehende Antikoagulation absetzen**
- ☐ **nur vorbestehende Antikoagulation fortführen**
- ☐ **Unfraktioniertes Heparin (UFH) PTT wirksam**
- ☐ **Unfraktioniertes Heparin (UFH) nicht PPT wirksam**
- ☐ **Niedermolekulares Heparin (NMH) mit Anti-Xa-Bestimmung**
- ☐ **Niedermolekulares Heparin (NMH) ohne Anti-Xa-Bestimmung**
- ☐ **Argatroban**
- ☐ **Fondaparinux**
- ☐ **Danaparoid**
- ☐ **Dabigatran**
- ☐ **Apixaban/Edoxaban oder Rivaroxaban**
- ☐ **Marcumar**
- ☐ **Keine mechanische Thromboseprophylaxe**
- ☐ **Intermittierende pneumatische Kompression (IPK)**
- ☐ **Medizinische Thromboseprophylaxestrümpfe (MTPS)**
- ☐ **nicht zutreffend**

zu Fall 1g): Bitte geben Sie, falls zutreffend, den Präparatenamen und die Dosierung bzw. den Präparatenamen und den Zielbereich an.

wie 1., jedoch zusätzlich:
h) Sepsis mit Nachweis von bakteriellen Erregern in der Blutkultur, mittlerer bis hoher Katecholaminbedarf UND DIC (livide Akren, Thrombozytenzahl 40.000 G/l, Quick < 50 %, D-Dimere stark erhöht, Fibrinogen < 100 mg/dl), aber keine Blutungssymptomatik.
(Mehrfachangaben sind möglich)

- ☐ **Keine Antikoagulation**
- ☐ **Unfraktioniertes Heparin (UFH) PTT wirksam**
- ☐ **Unfraktioniertes Heparin (UFH) nicht PPT wirksam**
- ☐ **Niedermolekulares Heparin (NMH) mit Anti-Xa-Bestimmung**
- ☐ **Niedermolekulares Heparin (NMH) ohne Anti-Xa-Bestimmung**
- ☐ **Argatroban**
- ☐ **Fondaparinux**
- ☐ **Danaparoid**
- ☐ **Keine mechanische Thromboseprophylaxe**
- ☐ **Intermittierende pneumatische Kompression (IPK)**
- ☐ **Medizinische Thromboseprophylaxestrümpfe (MTPS)**
- ☐ **nicht zutreffend**

zu Fall 1h): Bitte geben Sie, falls zutreffend, den Präparatenamen und die Dosierung bzw. den Präparatenamen und den Zielbereich an.

wie 1., jedoch zusätzlich:
i) Sepsis mit Nachweis von bakteriellen Erregern in der Blutkultur, mittlerer bis hoher Katecholaminbedarf besteht UND DIC (livide Akren, Thrombozytenzahl 40.000 G/l, Quick < 50 %, D-Dimere stark erhöht, Fibrinogen < 100 mg/dl), massive diffuse Blutungsneigung.
(Mehrfachangaben sind möglich)

- ☐ **Keine Antikoagulation**
- ☐ **Unfraktioniertes Heparin (UFH) PTT wirksam**
- ☐ **Unfraktioniertes Heparin (UFH) nicht PPT wirksam**
- ☐ **Niedermolekulares Heparin (NMH) mit Anti-Xa-Bestimmung**
- ☐ **Niedermolekulares Heparin (NMH) ohne Anti-Xa-Bestimmung**
- ☐ **Argatroban**
- ☐ **Fondaparinux**
- ☐ **Danaparoid**
- ☐ **Dabigatran**
- ☐ **Keine mechanische Thromboseprophylaxe**
- ☐ **Intermittierende pneumatische Kompression (IPK)**
- ☐ **Medizinische Thromboseprophylaxestrümpfe (MTPS)**
- ☐ **nicht zutreffend**

zu Fall 1i): Bitte geben Sie, falls zutreffend, den Präparatenamen und die Dosierung bzw. den Präparatenamen und den Zielbereich an.

Fall 2. Operativer Patient, 68 Jahre, keine kardiovaskulären Vorerkrankungen, abdominelle Sepsis, beatmet mit FiO2 0,45, kein bis niedriger Katecholaminbedarf (kein weiteres Organversagen, kein Schock), nach ausgedehnter fokussanierender abdomineller Operation, mit Blutverlust von ca. 1,5 l und stattgehabter Transfusion von allogenen Blutprodukten, Drainagen einliegend, aktuell keine aktive Blutung:
a) am 2. postoperativen Tag.
(Mehrfachangaben sind möglich)

- ☐ **Keine Antikoagulation**
- ☐ **Unfraktioniertes Heparin (UFH) PTT wirksam**
- ☐ **Unfraktioniertes Heparin (UFH) nicht PPT wirksam**
- ☐ **Niedermolekulares Heparin (NMH) mit Anti-Xa-Bestimmung**
- ☐ **Niedermolekulares Heparin (NMH) ohne Anti-Xa-Bestimmung**
- ☐ **Argatroban**
- ☐ **Fondaparinux**
- ☐ **Danaparoid**
- ☐ **Keine mechanische Thromboseprophylaxe**
- ☐ **Intermittierende pneumatische Kompression (IPK)**
- ☐ **Medizinische Thromboseprophylaxestrümpfe (MTPS)**
- ☐ **nicht zutreffend**

zu Fall 2a): Bitte geben Sie, falls zutreffend, den Präparatenamen und die Dosierung bzw. den Präparatenamen und den Zielbereich an.

wie 2., jedoch zusätzlich:
b) am 2. postoperativen Tag aber DIC, livide Akren, Thrombozytenzahl 40.000 G/l, Quick < 50 %, D-Dimere stark erhöht, Fibrinogen < 100 mg/dl, mittlerer bis hoher Katecholaminbedarf, keine Blutung.
(Mehrfachangaben sind möglich)

- ☐ **Keine Antikoagulation**
- ☐ **Unfraktioniertes Heparin (UFH) PTT wirksam**
- ☐ **Unfraktioniertes Heparin (UFH) nicht PPT wirksam**
- ☐ **Niedermolekulares Heparin (NMH) mit Anti-Xa-Bestimmung**
- ☐ **Niedermolekulares Heparin (NMH) ohne Anti-Xa-Bestimmung**
- ☐ **Argatroban**
- ☐ **Fondaparinux**
- ☐ **Danaparoid**
- ☐ **Keine mechanische Thromboseprophylaxe**
- ☐ **Intermittierende pneumatische Kompression (IPK)**
- ☐ **Medizinische Thromboseprophylaxestrümpfe (MTPS)**
- ☐ **nicht zutreffend**

zu Fall 2b): Bitte geben Sie, falls zutreffend, den Präparatenamen und die Dosierung bzw. den Präparatenamen und den Zielbereich an.

wie 2., jedoch zusätzlich:
c) am 2. postoperativen Tag aber DIC, livide Akren, Thrombozytenzahl 40.000 G/l, Quick < 50 %, D-Dimere stark erhöht, Fibrinogen < 100 mg/dl, mittlerer bis hoher Katecholaminbedarf, massive diffuse Blutungsneigung.
(Mehrfachangaben sind möglich)

- ☐ **Keine Antikoagulation**
- ☐ **Unfraktioniertes Heparin (UFH) PTT wirksam**
- ☐ **Unfraktioniertes Heparin (UFH) nicht PPT wirksam**
- ☐ **Niedermolekulares Heparin (NMH) mit Anti-Xa-Bestimmung**
- ☐ **Niedermolekulares Heparin (NMH) ohne Anti-Xa-Bestimmung**
- ☐ **Argatroban**
- ☐ **Fondaparinux**
- ☐ **Danaparoid**
- ☐ **Marcumar**
- ☐ **Keine mechanische Thromboseprophylaxe**
- ☐ **Intermittierende pneumatische Kompression (IPK)**
- ☐ **Medizinische Thromboseprophylaxestrümpfe (MTPS)**
- ☐ **nicht zutreffend**

zu Fall 2c): Bitte geben Sie, falls zutreffend, den Präparatenamen und die Dosierung bzw. den Präparatenamen und den Zielbereich an.

wie 2., jedoch zusätzlich:
d) am 2. postoperativen Tag, HIT Typ II in der Anamnese, Jahre zurückliegend, aktuell kein Hinweis auf thromboembolische Ereignisse oder Thrombozytenabfall.
(Mehrfachangaben sind möglich)

- ☐ **Keine Antikoagulation**
- ☐ **Unfraktioniertes Heparin (UFH) PTT wirksam**
- ☐ **Unfraktioniertes Heparin (UFH) nicht PPT wirksam**
- ☐ **Niedermolekulares Heparin (NMH) mit Anti-Xa-Bestimmung**
- ☐ **Niedermolekulares Heparin (NMH) ohne Anti-Xa-Bestimmung**
- ☐ **Argatroban**
- ☐ **Fondaparinux**
- ☐ **Danaparoid**
- ☐ **Dabigatran**
- ☐ **Apixaban/Edoxaban oder Rivaroxaban**
- ☐ **Keine mechanische Thromboseprophylaxe**
- ☐ **Intermittierende pneumatische Kompression (IPK)**
- ☐ **Medizinische Thromboseprophylaxestrümpfe (MTPS)**
- ☐ **nicht zutreffend**

zu Fall 2d): Bitte geben Sie, falls zutreffend, den Präparatenamen und die Dosierung bzw. den Präparatenamen und den Zielbereich an.

wie 2., jedoch zusätzlich:
e) am 2. postoperativen Tag bei akuter HIT Typ II, Thrombozytenabfall, Thrombosen, HIT-Schnelltest positiv.
(Mehrfachangaben sind möglich)

- ☐ **Keine Antikoagulation**
- ☐ **Unfraktioniertes Heparin (UFH) PTT wirksam**
- ☐ **Unfraktioniertes Heparin (UFH) nicht PPT wirksam**
- ☐ **Niedermolekulares Heparin (NMH) mit Anti-Xa-Bestimmung**
- ☐ **Niedermolekulares Heparin (NMH) ohne Anti-Xa-Bestimmung**
- ☐ **Argatroban**
- ☐ **Fondaparinux**
- ☐ **Danaparoid**
- ☐ **Dabigatran**
- ☐ **Apixaban/Edoxaban oder Rivaroxaban**
- ☐ **Keine mechanische Thromboseprophylaxe**
- ☐ **Intermittierende pneumatische Kompression (IPK)**
- ☐ **Medizinische Thromboseprophylaxestrümpfe (MTPS)**
- ☐ **nicht zutreffend**

zu Fall 2e): Bitte geben Sie, falls zutreffend, den Präparatenamen und die Dosierung bzw. den Präparatenamen und den Zielbereich an.

wie 2., jedoch zusätzlich:
f) am 2. postoperativen Tag bei DOAK als Vormedikation.
(Mehrfachangaben sind möglich)

- ☐ **Vorbestehende Antikoagulation absetzen**
- ☐ **nur vorbestehende Antikoagulation fortführen**
- ☐ **Unfraktioniertes Heparin (UFH) PTT wirksam**
- ☐ **Unfraktioniertes Heparin (UFH) nicht PPT wirksam**
- ☐ **Niedermolekulares Heparin (NMH) mit Anti-Xa-Bestimmung**
- ☐ **Niedermolekulares Heparin (NMH) ohne Anti-Xa-Bestimmung**
- ☐ **Argatroban**
- ☐ **Fondaparinux**
- ☐ **Danaparoid**
- ☐ **Dabigatran**
- ☐ **Apixaban/Edoxaban oder Rivaroxaban**
- ☐ **Marcumar**
- ☐ **Keine mechanische Thromboseprophylaxe**
- ☐ **Intermittierende pneumatische Kompression (IPK)**
- ☐ **Medizinische Thromboseprophylaxestrümpfe (MTPS)**
- ☐ **nicht zutreffend**

zu Fall 2f): Bitte geben Sie, falls zutreffend, den Präparatenamen und die Dosierung bzw. den Präparatenamen und den Zielbereich an.

wie 2., jedoch zusätzlich:
g) am 2. postoperativen Tag bei Marcumar als Vormedikation.
(Mehrfachangaben sind möglich)

- ☐ **Vorbestehende Antikoagulation absetzen**
- ☐ **nur vorbestehende Antikoagulation fortführen**
- ☐ **Unfraktioniertes Heparin (UFH) PTT wirksam**
- ☐ **Unfraktioniertes Heparin (UFH) nicht PPT wirksam**
- ☐ **Niedermolekulares Heparin (NMH) mit Anti-Xa-Bestimmung**
- ☐ **Niedermolekulares Heparin (NMH) ohne Anti-Xa-Bestimmung**
- ☐ **Argatroban**
- ☐ **Fondaparinux**
- ☐ **Danaparoid**
- ☐ **Dabigatran**
- ☐ **Apixaban/Edoxaban oder Rivaroxaban**
- ☐ **Marcumar**
- ☐ **Keine mechanische Thromboseprophylaxe**
- ☐ **Intermittierende pneumatische Kompression (IPK)**
- ☐ **Medizinische Thromboseprophylaxestrümpfe (MTPS)**
- ☐ **nicht zutreffend**

zu Fall 2g): Bitte geben Sie, falls zutreffend, den Präparatenamen und die Dosierung bzw. den Präparatenamen und den Zielbereich an.

Status quo Sepsis

Status quo Sepsis

Diesen Teil beantworten

Wie viele Patienten mit Sepsis / Septischen Schock werden jährlich in Ihrer Abteilung behandelt?

○ **<25** ○ **25-100** ○ **101-250** ○ **251-400** ○ **>400**

Welche Sepsisdefinition verwenden Sie im Alltag?

○ **SEPSIS-2 (Infektion und zwei oder mehr SIRS Kriterien / schwere Sepsis: Sepsis und Organversagen / septischer Schock: Sepsis und Kreislaufversagen)**

○ **SEPSIS-3 (Infektion und Anstieg des SOFA um zwei oder mehr 2 Punkte / Septischer Schock: Sepsis und Katecholaminbedarf und Serumlaktat 2mmol/l oder höher)**

○ **andere**

Führen Sie in der Notaufnahme bzw. auf den Normalstationen ihrer Einrichtung ein Screening anhand des quick SOFA durch?

○ **ja** ○ **nein**

Gibt es eine Standard Operating Procedure (SOP) zur Behandlung von Patienten mit Sepsis auf Ihrer Station?

○ **ja** ○ **nein**

Status quo Sepsis assoz. DIC

Status quo Sepsis assoz. DIC

Diesen Teil beantworten

Screenen Sie regelmäßig auf das Vorliegen einer Sepsis-assoziierten disseminierten intravasalen Koagulopathie (DIC)?

○ **ja mit JAAM-Score** ○ **ja mit ISTH-Score** ○ **ja mit anderem Score** ○ **nein**

Wenn Sie Anhaltspunkte für eine beginnende DIC haben, behandeln Sie diese?

○ **ja** ○ **nein**

Bitte geben Sie an, welche dieser Parameter/Devices bei Ihnen wie häufig gemessen bzw. genutzt werden:

1. Thrombozytenzahl

○ **täglich** ○ **ein- bis zweimal pro Intensivaufenthalt** ○ **bei speziellem Verdacht**

○ **theoretisch im Haus verfügbar**

2. Quick/INR

○ **täglich** ○ **ein- bis zweimal pro Intensivaufenthalt** ○ **bei speziellem Verdacht**

○ **theoretisch im Haus verfügbar**

3. aPTT

○ **täglich** ○ **ein- bis zweimal pro Intensivaufenthalt** ○ **bei speziellem Verdacht**

○ **theoretisch im Haus verfügbar**

4. ATIII

○ **täglich** ○ **ein- bis zweimal pro Intensivaufenthalt** ○ **bei speziellem Verdacht**
○ **theoretisch im Haus verfügbar**

5. D-Dimere

○ **täglich** ○ **ein- bis zweimal pro Intensivaufenthalt** ○ **bei speziellem Verdacht**
○ **theoretisch im Haus verfügbar**

6. Fibrinogen

○ **täglich** ○ **ein- bis zweimal pro Intensivaufenthalt** ○ **bei speziellem Verdacht**
○ **theoretisch im Haus verfügbar**

7. Protein C

○ **täglich** ○ **ein- bis zweimal pro Intensivaufenthalt** ○ **bei speziellem Verdacht**
○ **theoretisch im Haus verfügbar**

8. Protein S

○ **täglich** ○ **ein- bis zweimal pro Intensivaufenthalt** ○ **bei speziellem Verdacht**
○ **theoretisch im Haus verfügbar**

9. Faktor XIII

○ **täglich** ○ **ein- bis zweimal pro Intensivaufenthalt** ○ **bei speziellem Verdacht**
○ **theoretisch im Haus verfügbar**

10. TAT (thrombin-antithrombin-komplex)

○ **täglich** ○ **ein- bis zweimal pro Intensivaufenthalt** ○ **bei speziellem Verdacht**
○ **theoretisch im Haus verfügbar**

11. Prothrombinfragment F1,2

○ **täglich** ○ **ein- bis zweimal pro Intensivaufenthalt** ○ **bei speziellem Verdacht**
○ **theoretisch im Haus verfügbar**

12. Blutgasanalyse (inkl. pH, BE, Hb, Calcium, Laktat)

○ **täglich** ○ **ein- bis zweimal pro Intensivaufenthalt** ○ **bei speziellem Verdacht**
○ **theoretisch im Haus verfügbar**

13. Viskoelastisches Gerinnungsmonitoring (z.B. ROTEM, TEG)

○ **täglich** ○ **ein- bis zweimal pro Intensivaufenthalt** ○ **bei speziellem Verdacht**
○ **theoretisch im Haus verfügbar**

14. Aggregometrisches Gerinnungsmonitoring (z.B. Multiplate, ROTEM delta platelet, etc.)

○ **täglich** ○ **ein- bis zweimal pro Intensivaufenthalt** ○ **bei speziellem Verdacht**
○ **theoretisch im Haus verfügbar**

Bitte geben Sie in Stichpunkten Ihre Therapiestrategien bzw. Ihre Schwerpunkte bei der Therapie der DIC an.

Antikoagulation bei COVID-19

Antikoagulation bei COVID-19

Diesen Teil beantworten

Führen Sie bei zuvor gesunden Patienten mit COVID-19 generell eine andere medikamentöse Thromboseprophylaxe bzw. Antikoagulation durch als bei in der Krankheitsschwere vergleichbaren Patienten mit Sepsis?

○ **ja** ○ **nein**

Screenen Sie Patienten mit COVID-19 mit Hilfe eines Scores auf Koagulopathie?

○ **ja** ○ **nein**

Falls ja, mit welchem Score?

○ **ISTH** ○ **JAAM** ○ **anderer Score** ○ **nur D-Dimere** ○ **nicht zutreffend**

Wann beginnen Sie bei einem Patienten mit COVID-19 mit medikamentöser Thromboseprophylaxe bzw. Antikoagulation?

- ☐ **bereits auf Normalstation**
- ☐ **bei Aufnahme auf Intensivstation**
- ☐ **bei erhöhtem Score**
- ☐ **bei erhöhten D-Dimer-Werten**
- ☐ **bei nachgewiesener Thrombose**
- ☐ **nie**

Welches Strategie zur medikamentösen Thromboseprophylaxe bzw. Antikoagulation wenden Sie bei einem Patienten mit COVID-19 an (Fallbeispiel: invasive Beatmung, FiO2 0,8 , niedriger Katecholaminbedarf, erhöhte D-Dimer-Werte im Bereich 3-5 mg/l, kein Hinweis auf Thrombosen und/oder Embolien, kein Hinweis auf Blutung

- ☐ **Keine Antikoagulation**
- ☐ **Unfraktioniertes Heparin (UFH) PTT wirksam**
- ☐ **Unfraktioniertes Heparin (UFH) nicht PPT wirksam**
- ☐ **Niedermolekulares Heparin (NMH) mit Anti-Xa-Bestimmung**
- ☐ **Niedermolekulares Heparin (NMH) ohne Anti-Xa-Bestimmung**
- ☐ **Argatroban**
- ☐ **Fondaparinux**
- ☐ **Danaparoid**
- ☐ **Dabigatran**
- ☐ **Apixaban/Edoxaban oder Rivaroxaban**
- ☐ **Keine mechanische Thromboseprophylaxe**
- ☐ **Intermittierende pneumatische Kompression (IPK)**
- ☐ **Medizinische Thromboseprophylaxestrümpfe (MTPS)**
- ☐ **nicht zutreffend**

Freitext-Feld für Bemerkungen, Beobachtungen, Anregungen zu den Themen DIC/Thromboseprophylaxe/Antikoagulation bei COVID-19-Patienten

Wurde im Rahmen der Coronapandemie ein Screening mittels des qSOFA neu in Ihrem Haus etabliert?

○ **ja** ○ **nein**

Nutzen Sie einen anderen Screeningsscore zur Einschätzung des Schweregrades der Pneumonie (z.B. CRP-65)?

○ **ja** ○ **nein**

Abschicken
